# An improved, high-quality draft genome sequence of the Germination-Arrest Factor-producing *Pseudomonas fluorescens *WH6

**DOI:** 10.1186/1471-2164-11-522

**Published:** 2010-09-28

**Authors:** Jeffrey A Kimbrel, Scott A Givan, Anne B Halgren, Allison L Creason, Dallice I Mills, Gary M Banowetz, Donald J Armstrong, Jeff H Chang

**Affiliations:** 1 Department of Botany and Plant Pathology, Oregon State University, Corvallis, OR 97331, USA; 2 Molecular and Cellular Biology Program, Oregon State University, Corvallis, OR 97331, USA; 3 Center for Genome Research and Biocomputing, Oregon State University, Corvallis, OR 97331, USA; 4 USDA-ARS NFSPRC National Forage Seed Production Research Center, 3450 S. W. Campus Way, Corvallis, OR 97331, USA; 5 Informatics Research Core Facility, University of Missouri, Columbia, MO 65211 USA

## Abstract

**Background:**

*Pseudomonas fluorescens *is a genetically and physiologically diverse species of bacteria present in many habitats and in association with plants. This species of bacteria produces a large array of secondary metabolites with potential as natural products. *P. fluorescens *isolate WH6 produces Germination-Arrest Factor (GAF), a predicted small peptide or amino acid analog with herbicidal activity that specifically inhibits germination of seeds of graminaceous species.

**Results:**

We used a hybrid next-generation sequencing approach to develop a high-quality draft genome sequence for *P. fluorescens *WH6. We employed automated, manual, and experimental methods to further improve the draft genome sequence. From this assembly of 6.27 megabases, we predicted 5876 genes, of which 3115 were core to *P. fluorescens *and 1567 were unique to WH6. Comparative genomic studies of WH6 revealed high similarity in synteny and orthology of genes with *P. fluorescens *SBW25. A phylogenomic study also placed WH6 in the same lineage as SBW25. In a previous non-saturating mutagenesis screen we identified two genes necessary for GAF activity in WH6. Mapping of their flanking sequences revealed genes that encode a candidate anti-sigma factor and an aminotransferase. Finally, we discovered several candidate virulence and host-association mechanisms, one of which appears to be a complete type III secretion system.

**Conclusions:**

The improved high-quality draft genome sequence of WH6 contributes towards resolving the *P. fluorescens *species, providing additional impetus for establishing two separate lineages in *P. fluorescens*. Despite the high levels of orthology and synteny to SBW25, WH6 still had a substantial number of unique genes and represents another source for the discovery of genes with implications in affecting plant growth and health. Two genes are demonstrably necessary for GAF and further characterization of their proteins is important for developing natural products as control measure against grassy weeds. Finally, WH6 is the first isolate of *P. fluorescens *reported to encode a complete T3SS. This gives us the opportunity to explore the role of what has traditionally been thought of as a virulence mechanism for non-pathogenic interactions with plants.

## Background

*Pseudomonas fluorescens *is a diverse species of bacteria that is found throughout natural habitats and associated with plants. Contributing to their diverse lifestyles is their ability to produce an equally diverse array of secondary metabolites that affect interactions with hosts and other inhabitants of their ecosystems. Some isolates benefit plants by producing growth promoting hormones or antimicrobial compounds to control against pathogens [1]. Others are deleterious and have the capacity to synthesize and secrete novel compounds that negatively affect growth of plants [2-4].

The physiological diversity of *P. fluorescens *is mirrored by its tremendous genetic diversity. However, the genetic diversity may reflect the possibility that *P. fluorescens *is not a single species, but rather a complex of at least two lineages. Molecular phylogenetic studies of 16 isolates suggested *P. fluorescens *should be represented by the *P. chlororaphis *and *P. fluorescens *lineages [5]. Alternatively or additionally, *P. fluorescens *may have an open pan genome [6,7]. Finished genome sequences are available for the SBW25, Pf-5, and Pf0-1 isolates of *P. fluorescens *[8,9]. Their genomes exceed 6.4 megabases and their relatively large sizes are not unexpected for free-living bacteria [10]. Comparative analyses of the three isolates of *P. fluorescens *revealed substantial variation in diversity of genome content and heterogeneity in genome organization [9]. Each genome has 1,000 to nearly 1,500 unique genes when compared to each other.

Plant-associated isolates of *P. fluorescens *potentially have mechanisms for interacting with plants. Many Gram-negative bacteria use a type III secretion system (T3SS) to interact with their hosts [11]. The T3SS is the most complex of the bacterial secretion systems and is typically encoded by a large cluster of genes arranged as a single superoperon. Its function is to inject type III effector proteins directly into host cells [11,12]. These type III effectors are important host-range determinants of plant pathogenic bacteria because they perturb and potentially elicit plant defenses [13].

It is unclear as to how prevalent T3SS-encoding regions are in *P. fluorescens*. Nearly 60% of a surveyed collection of *P. fluorescens *strains had a homolog of *rscN*, which encodes the ATPase of the T3SS [14]. However, it is not known whether all genes necessary to complete the T3SS are present in these isolates. Of the three completed genomes, genes encoding the T3SS are present only in SBW25. Several important or typically conserved genes are missing or truncated in the T3SS-encoding locus of SBW25 [15]. Despite the cryptic appearance of the T3SS, when constitutively expressing the transcriptional regulator of the T3SS, SBW25 could deliver a heterologous type III effector into plant cells, suggesting the T3SS may still be functional [15].

The role of the T3SS for the lifestyle of *P. fluorescens *is still unclear. In SBW25, despite the cryptic T3SS, single mutants of some but not all the remaining T3SS-encoding genes were reduced in fitness in the rhizosphere of sugar beets [16]. This is not unheard of, as mutants of seemingly cryptic T3SS in pathogens are compromised in virulence [17]. However, in the case of SBW25, the T3SS mutants were also compromised in growth *in vitro *[16]. A T3SS mutant of the biocontrol isolate *P. fluorescens *KD was compromised in its ability to protect cucumbers against damping-off disease caused by *Pythium ultimum *[18]. This may be a result of KD requiring a functional T3SS to elicit host defenses, thereby indirectly protecting against *P. ultimum *or potentially as a direct mechanism against the pathogen.

We are interested in exploiting *P. fluorescens *for control of grassy weeds. We have previously reported the selection, isolation, and characterization of five strains of *P. fluorescens *that inhibit germination of seeds of grassy weeds [19]. Further characterizations led to the identification of Germination-Arrest Factor (GAF) produced by these isolates. GAF is a small, extremely hydrophilic secreted herbicide that reacts with ninhydrin and possesses an acid group, suggestive of a small peptide or amino acid analog [4,20]. In particular, the high specificity of GAF towards grasses and inhibitory activity at only certain developmental stages during seed germination provides promise for its potential as a natural herbicide for the control of grassy weeds in grass seed production and turf management settings.

We selected *P. fluorescens *WH6 as the archetypal GAF-producing isolate. WH6 was extracted from the rhizosphere of *Poa *sp. and *Triticum aestivum *at the Hyslop Research Farm in Benton County, Oregon, USA [19]. We sequenced and developed an improved high-quality draft sequence for WH6 using a hybrid Illumina and 454-based sequencing approach. This standard is considered sufficient for our purposes of assessing gene inventory and comparing genome organization [21].

Comparative genomic analysis showed a high number of orthologous genes and strong similarity in genome organization between WH6 and SBW25. Phylogenomic analysis supported this observation and placed WH6 in the same lineage as SBW25, or the proposed *P. fluorescens *lineage. The high similarity in orthology and genome organization is in contrast to previous observations of *P. fluorescens *and in comparisons of WH6 to Pf-5 or Pf0-1 [9]. From a non-saturating Tn*5*-mutagenesis screen of WH6, we previously identified two mutants compromised in GAF activity (WH6-2::Tn*5 *and WH6-3::Tn*5*; [4]). Mapping of DNA sequences flanking the two mutants revealed genes encoding proteins with potential functions in regulation and biosynthesis of GAF. Finally, inspection of the WH6 genome revealed several candidate host-association mechanisms, including what appears to be a complete type III and two complete type VI secretion systems.

## Results and Discussion

### Sequencing and developing an improved, high-quality draft genome sequence

We used an Illumina and a 454 FLX GS LR70 to sequence the genome of WH6 (Table 1). The theoretical coverage using all filtered reads was estimated to be 316× assuming a genome size of approximately 6.5 megabases. We employed a number of steps to meet the standards of an improved, high-quality draft genome sequence of WH6 for comparative purposes. We used Velvet 0.7.55, combinations of short-reads, and a variety of parameter settings to *de novo *assemble the short reads to generate approximately 75 different assemblies [22]. We developed and used *ad hoc *Perl scripts with an associated visualization tool to compare each of the different assemblies to each other. This step enabled us to eliminate entire assemblies with large contigs not supported by any other assembly.

**Table 1:** High-throughput sequencing statistics

Method	Total Reads	Reads Used	Theoretical Coverage*	# Contigs (>100 bp)^	**Total size (Mb)**^**§**^
Sanger	178	178	n/a	n/a	n/a

GAI 32 single	16,852,820	9,298,356	83	5,884	6.06

GAII 70 PE	10,854,745	9,013,849	234	95	6.27

454	202,070	200,467	7	2,204	6.10

All short reads	38,764,380	23,742,926	316	256	6.26

All reads	38,764,558	23,810,966	316	115	6.27

*Based on approximated genome size of 6.5 Mb; ^Highest confident draft assemblies using Velvet 0.7.55 [22]; ^Improved, high quality draft including Sanger reads. n/a = not applicable; ^§^Based on sum total of all contigs > 100 bp in length.

We identified a single high-quality *de novo *assembly based on nearly 24 million reads from all three sequencing methods (Table 1). The Velvet parameters were hash length of 31, expected coverage of 104, and a coverage cutoff of 20. Actual coverage of this assembly based on the total number of used reads was 65 ~ 120×, which was less than one-third the theoretical coverage. This assembly had 189 contigs greater than one kb and a total of 256 contigs greater than 100 bp. The largest contig was 264 kb and the N50 number and size were 26 contigs and 78 kb, respectively.

We used experimental and *in silico *approaches to improve the draft assembly by reducing the number of physical gaps. Of the 189 contigs greater than one kb, 139 contigs (74%) had significant homology to a reference sequence shared by the end of another contig. These 139 contigs potentially flanked 111 physical gaps (See Additional file 1: Table S1). We were able to amplify across 86 (77.5%) of the gaps using PCR. Physical gaps were subsequently resolved by reassembling the nearly 24 million short-reads with the 86 Sanger reads. Of the remaining scaffolds, we associated more based on *in silico *evidence. Some contigs shared long-range synteny to a reference genome (see below) and their ends had fifteen or more basepairs of sequence with 100% overlap to each other. This phenomenon is a result of Velvet failing to extend the contig because of low coverage. Secondly, some contigs could be paired together because their ends had partial coding regions with homology to a common reference gene. In total, nineteen more contigs were associated, resulting in a final assembly of 115 scaffolds greater than 100 bp. The largest scaffold was 814 kb and the N50 number and size were 8 and 203 kb, respectively.

The improved, high-quality draft genome sequence had 67 sequence gaps totaling 258,650 Ns. There were 45 large sequence gaps with more than 300 Ns of which eight had more than 10,000 Ns each. We presumed these were artifacts of the Velvet assembly because the fragment size of our paired-end library was no larger than 300 bp. We corrected the sizes for 31 gaps to their corresponding length found in homologous reference sequences. In the other 14 cases, we simply reduced the number of Ns in the region to 300 bp, to reflect the maximum size of our paired-end library. Both approaches to correct the size of sequence gaps were validated using PCR of randomly selected regions (data not shown). In total, we reduced the number of Ns to 6,049 or ~2% of the original number of Ns.

The release of the finished genome sequence of SBW25 fortuitously coincided with our efforts of improving the draft genome sequence of WH6 [9]. We noted that nearly 90% of the homologous sequences we found in the NCBI nt dataset using our BLASTN-based approaches were to *P. fluorescens *SBW25. We therefore surmised that the genome of WH6 would be similar to the finished genome of *P. fluorescens *SBW25 and used it as a reference for Mauve Aligner to reorder the 115 WH6 scaffolds [9,23].

**Figure 1:**
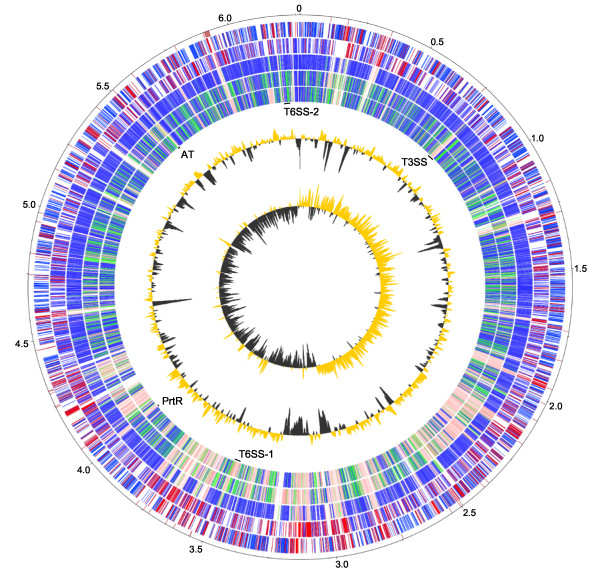
**Circular representation of the improved, high-quality draft genome sequence of WH6**. The outer scale designates the coordinates in half million base pair increments. The red ticks indicate physical gaps. Circles 2 and 3 show the predicted coding regions of WH6 on the positive and negative strands, respectively. Coding regions are colored to highlight orthologous (blue) and 1567 unique (red) coding regions of WH6 relative to the other sequenced *P. fluorescens*. Circles 4, 5, and 6 show orthologs (BLASTP e-value ≤ 1 × 10^-7^) of SBW25, Pf-5, and Pf0-1, respectively. The extent of homology relative to WH6 is depicted using a heat map of arbitrarily chosen bins; dark blue: orthologs with greater than 80% homology over the length of the gene; green: orthologs with between 60-80% homology over the length of the gene; pink: orthologs with between 20-60% homology over the length of the gene; white: no homology (less than 20% homology over the length of the gene). The positions of loci of interest are also denoted (see corresponding text for more details). Circles 7 and 8 show GC% (gold >60.6% average; gray < 60.6% average) and GC-skew.

The genome of WH6 is presumed to be a single circular chromosome (Figure 1). A total of 53 scaffolds greater than one kb could be ordered using Mauve Aligner. The remaining 62 contigs could not be reordered and were excluded from our circular representation of the genome. These 62 contigs were all smaller than one kb and their sum total was only 13 kb. Attempts to use Pf0-1 or Pf-5 as a reference for Mauve Aligner were largely unsuccessful, supporting our observation that WH6 and SBW25 had higher synteny than previously detected in *P. fluorescens *and suggesting our WH6 *de novo *assembly was of high quality. We found no evidence of plasmids in the genome of WH6.

This Whole Genome Shotgun project has been deposited at DDBJ/EMBL/GenBank under the accession AEAZ00000000. The version described in this paper is the first version, AEAZ01000000. The WH6 genome sequence and its associated tools can also be accessed from our website at: http://changbugs.cgrb.oregonstate.edu/microbes/org_detail.html?org=WH6-G3.

One challenge with *de novo *assembly is dealing with repeated sequences [24]. Small repeated sequences are present in genomes of *P. fluorescens *but were not expected to have a large effect on our ability to assemble the WH6 genome because of the size of our paired-end fragments [9]. Larger repeats, however, could not be resolved. We only observed one rRNA operon in the genome of WH6. We suspect that WH6 has five rRNA operons similar to SBW25 and Pf-5, but they collapsed into one contig. There was approximately 5× more coverage for the contig containing the one rRNA operon of WH6 compared to the other contigs. Similarly, nonribosomal peptide synthases (NRPSs) are encoded by large genes with repeated modules [25,26]. The modular domains either collapsed on one another in the assembly, or were assembled into short contigs that we could not extend. A large fraction of these partial NRPS-encoding genes were found in the small contigs that we could not reorder using Mauve Aligner. Here too, we noticed higher coverage than the other scaffolds.

### Comparative and phylogenomic analyses of *P. fluorescens*

At a large scale, the genome of WH6 was similar to the genomes of the other *P. fluorescens *isolates (Table 2). The size of the genome is slightly smaller, which may be a consequence of the draft nature of our genome assembly. Nonetheless, the 5876 predicted coding sequences (CDSs) and 89.2% coding capacity were very similar.

**Table 2:** Comparison of *P. fluorescens *genome characteristics

Isolate	WH6*	SBW25^	**Pf-5**^**§**^	Pf0-1^
Genome Size	6.27 Mb	6.72 Mb	7.07 Mb	6.44 Mb

GC %	60.6	60.5	63.3	60.5

Coding regions	5876	5921	6138	5736

Avg. length of coding sequences	951	1000	1020	1006

Coding %	89.2	88.1	88.5	89.6

*Improved, high quality draft genome sequence ^[9]; ^§^[8].

**Figure 2:**
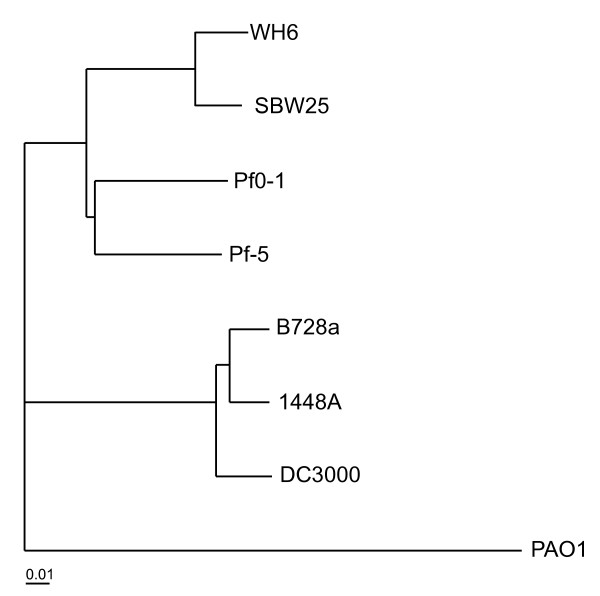
**Phylogenomic tree of eight *Pseudomonas *isolates based on a super alignment of 1966 translated sequences**. *P. fluorescens *isolates: WH6, SBW25, Pf-5, and Pf0-1; *P. syringae *pathovars: *tomato *DC3000, *phaseolicola *1448A, and *syringae *B728a; *P. aeruginosa *PAO1. Bootstrap support for nodes (r = 1000) were all 100. The scale bar indicates the number of amino acid substitutions per site.

Previous analyses of *P. fluorescens *found SBW25, Pf-5, and Pf0-1 to be divergent, with only ~61% of the genes shared and little long-range synteny [9]. We used HAL to carry out similar analyses to determine the effect of the WH6 genome on the phylogenetic relationship of the *P. fluorescens *species and potential changes to the size of its pan genome [27]. HAL uses a Markov Clustering algorithm based on e-values from reciprocal all-by-all BLASTP analysis to create clusters of orthologs. Core sequences from each species are concatenated and the super alignment is used in phylogenomic analysis. Using a core of 1966 translated sequences common to *P. fluorescens*, representative strains of *P. syringae*, and *P. aeruginosa *PAO1, HAL clustered the different species of *Pseudomonas *as expected [8,9,28-30]. Further, HAL clearly defined two separate lineages for *P. fluorescens*, placing WH6 with SBW25 (Figure 2).

**Figure 3:**
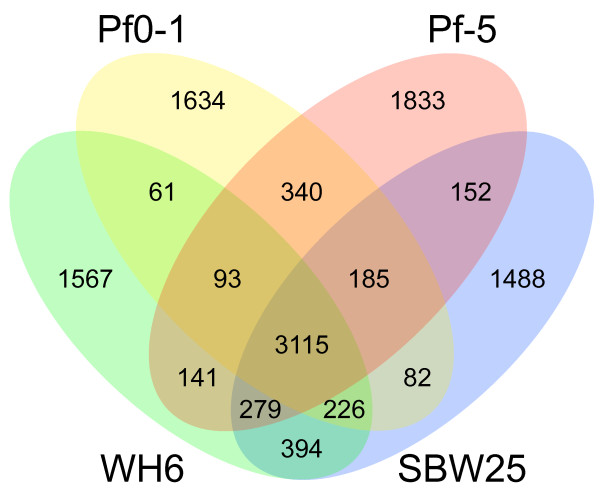
**Venn diagram comparing the gene inventories of four isolates of *P. fluorescens***. The numbers of shared and unique genes are shown. Comparisons were made using HAL.

**Figure 4:**
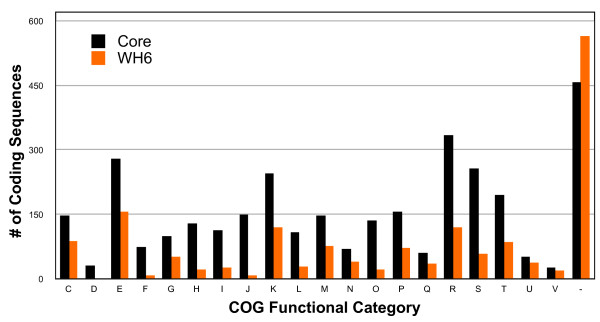
**Functional categories of the 3115 core genes of *P. fluorescens *and 1567 unique genes of WH6**. The number of genes in each category are presented above each bar; black = core; orange = WH6. Categories: **C) **energy production and conversion; **D) **cell cycle control, cell division, chromosome partitioning; **E) **amino acid transport and metabolism; **F) **nucleotide transport and metabolism; **G) **carbohydrate transport and metabolism; **H) **coenzyme transport and metabolism; **I) **lipid transport and metabolism; **J) **translation, ribosomal structure and biogenesis; **K) **transcription; **L) **replication, recombination and repair; **M) **cell wall/membrane/envelope biogenesis; **N) **cell motility; **O) **posttranslational modification, protein turnover, chaperones; **P) **inorganic ion transport and metabolism; **Q) **secondary metabolites biosynthesis, transport and catabolism; **R) **general function; **S) **unknown function; **T) **signal transduction mechanisms; **U) **intracellular trafficking, secretion and vesicular transport; **V) **defense mechanisms; **-) **no COG designation.

Within the *P. fluorescens *species as presently defined, 3115 genes formed the core and represented 53%, 52.6%, 50.7%, and 54.3% of the genomes of WH6, SBW25, Pf-5, and Pf0-1, respectively (Figure 3). This was nearly a 10% reduction relative to previous analysis of three genomes [9]. A large fraction of the core genes was assigned to categories with general cellular processes such as energy production and conversion, amino acid transport and metabolism, translation, and transcription (Figure 4). Approximately 90% of the 3115 core genes clustered with orthologs sharing identical COG designations suggesting our automated annotation pipeline was accurate. There were some exceptions but their rarity and subtle differences did not warrant manual curation. For example, one cluster of orthologs had genes annotated as "arabinose efflux permeases" (COG2814) for genes from the published isolates of *P. fluorescens *but "permease of the major facilitator superfamily" (COG0477) for the ortholog of WH6.

A total of 4309 of the translated products of WH6 had an orthologous sequence in another isolate of *P. fluorescens*. Almost 69% of the WH6 genes had an orthologous sequence in SBW25, as compared to Pf-5 and Pf0-1 with 62% and 59%, respectively (Figures 1 and 3). We found similar levels of overlap using reciprocal BLASTP (data not shown). The 69% orthology between WH6 and SBW25 is much higher than previously observed between isolates of *P. fluorescens *[9]. These levels were still lower than those between different pathovars of *P. syringae*, which had greater than 80% orthology [29,31,32]. Therefore, the generalization that *P. fluorescens *have highly variable genomes still holds true.

**Figure 5:**
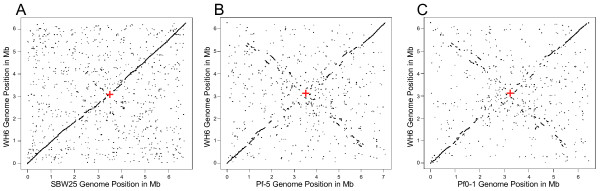
**Synteny plots comparing the organization of the WH6 genome to that of the three other isolates of *P. fluorescens***. Unique 25 mers from *P. fluorescens *isolates SBW25 **(A)**, Pf-5 **(B)**, and Pf0-1 **(C) **were compared to the improved, high quality draft genome sequence of WH6. The start positions of all matching pairs were plotted in an XY graph with the coordinates of the genomes of SBW25, Pf-5, and Pf0-1 along the x-axis and coordinates of the genome of WH6 along the y-axis. The termini are located at the approximate mid-way point for each comparison (red **+**). Genome scales are shown in one Mb increments.

The genomes of WH6 and SBW25 also showed extensive long-range synteny (Figure 5). This amount of synteny was unexpected given previous comparisons [9]. When compared to Pf-5 or Pf0-1, we found little long-range synteny, which tended to be near the origin of replication. Synteny rapidly degraded away from the origin with an increase in inversions between the genomes [9]. Taken together these lines of evidence all suggest WH6 and SBW25 to be similar and support, though perhaps prematurely, a redefinition of the *P. fluorescens *species [5,9].

It could be argued that the high level of synteny we found with SBW25 was an artifact of using SBW25 to reorder the WH6 scaffolds. Though we cannot exclude this possibility, we highlight several points that suggest otherwise. We used a *de novo *approach to assemble the genome of WH6. The long-range synteny to the SBW25 genome was observed within each and across the *de novo *assembled scaffolds of WH6 (Figure 5). Furthermore, synteny with SBW25 was also supported by our ability to use SBW25 to successfully and substantially reduce the number of WH6 scaffolds and improve the WH6 genome sequence (Figure 1). Finally, analysis of GC skew gave higher confidence in the reordering of WH6 scaffolds (Figure 1, track 8). Genomes often have a bias of guanine in the leading strand [33,34]. Inversions of GC skew in regions distant from the replication origins and termini are indicative of a recent recombination event [35]. Barring these events, inversions of GC skew could also potentially indicate large-scale misassemblies or incorrect reordering of contigs. For the most part, the genome of WH6 showed the expected bias of guanine in the leading strand; there are perhaps two small inversions in GC skew flanked by physical gaps between scaffolds near the terminator. Our use of SBW25 as a reference for reordering scaffolds is therefore acceptable and the observed synteny between WH6 and SBW25 appeared to reflect true similarities in genome organization.

More than 30% of the WH6 coding regions were unique (Figures 1 and 3). Examinations of their annotated functions suggested greater diversity in metabolic and host-association functions such as carbohydrate transport and metabolism, inorganic ion transport and metabolism, secondary metabolite biosynthesis, transport and catabolism, intracellular trafficking, secretion and vesicular transport, as well as defense mechanisms (Figure 4).

Examples of CDSs unique to WH6 and enriched in these functional categories include 35 candidate permeases of the major facilitator superfamily, a large and diverse superfamily of secondary active transporters that control movement of substrates across membranes (COG0477; [36]). WH6 also had 12 unique CDSs that encode for putative TonB-dependent receptors, involved in uptake of iron and potentially other substrates (COG1629; [37]; see also section entitled, "Regulators of gene expression"). Restriction modification (RM) systems are widespread defense mechanisms that protect prokaryotes from attack by foreign DNA [38]. RM systems are diverse and can vary dramatically in numbers. WH6 has at least 30 CDSs with annotated functions or domains common to proteins of RM systems. PFWH6_5037-5039, for example, encode for a type I RM system that appears to be unique to WH6. Finally, other CDSs unique to WH6 and of direct interest to us are described in the following sections. The greater than 1500 genes unique to WH6 were dispersed throughout its genome with only a slight bias in location closer to the terminators (Figure 1). This bias was previously generalized for *P. fluorescens *[9].

### Mapping GAF mutants

We previously identified two WH6 mutants from a non-saturating Tn*5*-mutagenesis screen for those affected in arresting the germination of *Poa *seeds [4]. We cloned, sequenced and mapped their flanking sequences to identify the disrupted genes. Mutant WH6-3::Tn*5 *had an insertion in PFWH6_3687. This CDS is annotated as a "predicted transmembrane transcriptional regulator (anti-σ factor)". Its closest homolog, with 94% similarity is PrtR encoded by *P. fluorescens *LS107d2 [39]. The Tn*5 *element had inserted at nucleotide position 417 within codon Asp139. Because loss of *prtR *led to a loss of GAF activity, PrtR is likely an activator rather than a repressor, as was the case in *P. fluorescens *LS107d2 [39].

Just upstream of *prtR *in WH6 is *prtI*, which encodes a candidate ECF σ^70 ^factor. This arrangement is reminiscent of many sigma-anti-sigma factor pairs and suggests that the genes are potentially co-regulated and both may have roles in regulating GAF gene expression [40]. It is peculiar that we failed to identify an insertion in *prtI *but one obvious explanation is that our screen was not saturating. Regardless, it will be important to examine the necessity of PrtI for GAF activity to resolve its role.

Mutant WH6-2::Tn*5 *had an insertion in PFWH6_5256, a gene encoding a candidate aminotransferase class III. The identification of an aminotransferase as necessary for GAF supports our previous findings suggesting that GAF contains an amino group and may be a small peptide or amino acid analog [4]. Aminotransferases are pyridoxal phosphate (PLP)-dependent enzymes that catalyze the transfer of an amino group from a donor group (commonly an amino acid) to an acceptor molecule [41]. The Tn*5 *element had inserted at nucleotide position 1124 within codon Lys375. Based on comparisons to the acetyl ornithine aminotransferase family the insertion is distal to the conserved residues that compose pyridoxal 5'-phosphate binding sites, the conserved residues that compose inhibitor-cofactor binding pockets, and the catalytic residue [42]. Further characterization of WH6-2::Tn*5 *is necessary to examine its enzymatic properties and role in biosynthesis of GAF.

### Regulators of gene expression

Bacteria with large genomes tend to have complex regulatory networks to integrate and respond to a multitude of environmental signals. The extracytoplasmic function (ECF) σ^70 ^factors are a class of important transcriptional regulators of cell-surface signaling systems. Using a Hidden Markov Model (HMM) for ECF-encoding genes, we found 19, 26, 28, and 22 ECFs in WH6, SBW25, Pf-5 and Pf0-1, respectively [43]. Of the 19 identified in WH6, ten are part of the core set common to all four sequenced *P. fluorescens *isolates and included *prtI *and *prtR*, which we identified as necessary for GAF activity. Because we had previously shown that Pf-5 and Pf0-1 do not have GAF activity, these results suggest that the putative PrtI/R-regulon may be different between the different isolates of *P. fluorescens *[4]. Four of the 19 ECF-encoding genes were exclusive to the plant-associated strains WH6, SBW25, or Pf-5. Two of these were only shared with SBW25, of which one was *rspL *(see below). The other two lacked sufficient annotations to speculate on their functions. The remaining five ECFs were unique to WH6 and all are potentially co-expressed with genes encoding outer membrane receptors involved in iron perception or uptake (*chuA, fhuA*, and *fhuE*).

### Virulence factors

Pseudomonads produce a wide-range of secondary metabolites with potential benefit or detriment to plants and microbes [25,44]. Many are synthesized by non-ribosomal peptide synthases (NRPS) or polyketide synthases [25,26,44]. We found evidence for several NRPS-encoding genes. Because of their modular architecture, most NRPS-encoding genes of WH6 were fragmented and found on small contigs that failed to assemble or reorder. Therefore, it was not possible to determine the structure of the repeats or infer functions based on homology. We were, however, able to identify several other candidate toxins and virulence factors (Table 3).

**Table 3:** Candidate Host-association and virulence factors*

WH6 Gene (PFWH6_)	Host-association or virulence factor	Reported function	Reference
0718-0737	T3SS-encoding region	Host-association; secretion apparatus	[15,84]

5796-5812	T6SS-1-encoding region	Host-association; secretion apparatus	[68,69,73]

3251-3270	T6SS-2-encoding region	Host-association; secretion apparatus	[68,69]

0824-0827	Betaine/choline uptake	Osmoprotection	[85]

5455 & 0252	BCCT Transporter	Choline transport	[86]

5456-5458	Choline to Betaine	Choline to Betaine conversion	[7]

1723 & 2895	*aprA*^	Alkaline protease A; Insecticidal toxin	[87]

4097/98, 4099, 4100	*tca-d*	Insecticidal toxin	[88]

5264, 5082, 3503 & 0070	*katA*	Catalase A; H_2_O_2 _protection	[89]

0985 - 0996	Synthesis of Alginate	Exopolysaccharide	[90]

2199	Synthesis of Levansucrase^	Exopolysaccharide	[91,92]

4225	*marR*	Transcriptional regulator virulence factors	[93]

3833 - 3843	T2SS-encoding region	Secretion apparatus	[94]

0699	*plc *lipase	Phospholipase C; virulence factor	[95]

0396 - 0398	*TAT-encoding region	Secretion apparatus	[96,97]

4428, 2727, 0116-0120	Synthesis of mangotoxin	Antimetabolite toxin	[98]

2331-2334	Synthesis of hydrogen cyanide	Inhibitor of cytochrome c oxidase	[25]

*Table derived from [44]; candidates were identified using BLASTP (e-value 1 × 10^-7^). There are 43 WH6 proteins with homology to candidate TAT-secreted proteins of *P. syringae *pv *tomato *DC3000 [96,97]; ^no orthologs were detected in genomes of other *P. fluorescens*.

**Figure 6:**
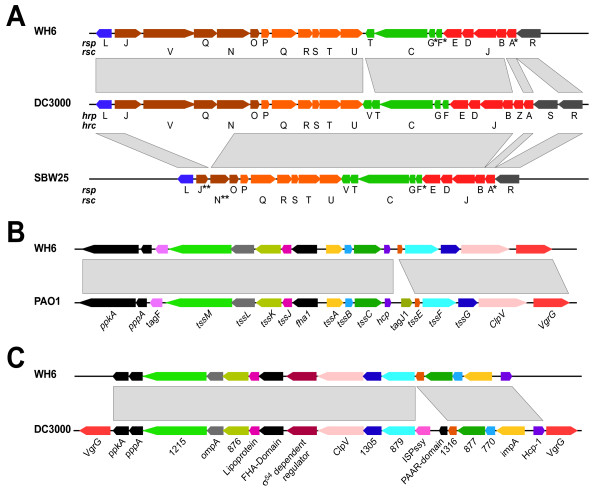
**Schematic Representations and Comparisons of Type III and Type VI Secretion Systems**. **A) **The type III secretion system of WH6 (top) compared to that of *P. syringae *(middle), and SBW25 (bottom). Co-transcribed genes and orthologous transcriptional units are colored similarly. *No detectable homology but inferred based on location in the T3SS-encoding locus. **Truncated coding regions. **B) **The type VI secretion system-1 of WH6 (top) compared to HSI-I of *P. aeruginosa *PAO1. **C) **The type VI secretion system-2 of WH6 (top) compared to a candidate type VI secretion system of *P. syringae *pv *tomato *DC3000 (bottom). Orthologous transcriptional units are colored similarly. For **A-C**, the directions of transcription are represented. Gray boxes highlight homologous regions.

We identified several secretion systems in WH6 unique to host-associated bacteria and/or necessary for full virulence of pathogenic bacteria. WH6 appears to encode a complete and functional type III secretion system (PFWH6_0718-0737; Figure 6a). We named its genes according to the nomenclature first proposed for SBW25 [15,45]. There is strong homology and synteny between the T3SS-encoding regions of WH6 and *P. syringae*, raising the possibility of a recent acquisition of the T3SS-encoding locus by WH6, similar to KD [14]. Phylogenetic analyses of *rscN*, however, placed WH6 with the group 8 of biocontrol isolates of *P. fluorescens *(data not shown; [14]). Additionally, 15 kb of sequences on either side of the T3SS-encoding region of WH6 were highly syntenic to regions flanking the T3SS-encoding region of SBW25 with the exception of the type III effector gene, *ropE*. Together, these data argue against a recent acquisition of the T3SS-encoding region by WH6.

There were some differences between T3SS-encoding regions of WH6 and *P. syringae*. The *rspR, rspZ*, and *rspV *genes of WH6 were not present and we failed to detect any homology between the *rspF/hrpF, rspA/hrpA*, and *rspG/hrpG *genes. Data, however suggest these differences likely have little to no effect on T3SS function. HrpR and HrpS are highly similar and are functionally redundant. In some *Erwinia *strains, HrpS by itself is demonstrably sufficient for T3SS function [46,47]. Deletion mutants of *hrpZ *are still functional and HrpV functions as a negative regulator of the T3SS [48-50]. HrpF and HrpA are homologous to each other and are structural components of the T3SS. They are the most polymorphic proteins encoded within the T3SS-cluster and the absence of significant homology between *rspF *and *rspA *to their counterparts of *P. syringae *was therefore not surprising [51,52]. Our automated annotation approach failed to identify *rspG *but upon visual inspection, we noted a small CDS that encodes a potential product of 63 amino acids. BLAST searches failed to detect homology to *hrpG*, but given its position in the T3SS-encoding region and similarity in size to the translated product of *hrpG*, we have annotated it as *rspG*. In total, these data support the notion that WH6 encodes a complete and functional T3SS, although, its role in the lifestyle of WH6 remains unknown.

### Candidate type III effectors of WH6

We used a homology-based approach to search for type III effector genes in the genome of WH6. Our database of type III effectors included those from T3SS-using phytopathogens and some mammal pathogens. We only identified one translated sequence with homology to PipB from *Salmonella*, and another with homology to HopI1 from *P. syringae *(e-value < 1 × 10^-7^, > 33% identity; [53]). However, neither appeared to be strong candidates for a type III effector. We identified a homolog of *pipB *in the genome of Pf-5, which does not encode the T3SS. HopI1 encodes a J domain and its homolog in WH6 was annotated as the molecular chaperone, *dnaJ *[54]. These results suggest that if WH6 does encode type III effectors, they are very divergent in sequence. SBW25, in contrast, had at least five genes with homology to known type III effectors, of which two were expressed to sufficiently high levels and delivered by a heterologous T3SS-encoding bacterium [55].

Computational approaches have been successfully used to identify candidate type III effectors from *P. syringae*, based in part on identifying a cis-regulatory element upstream of their genes and also some genes of the T3SS [56-58]. This so-called *hrp*-box is recognized by HrpL, an extracytoplasmic function (ECF) σ^70 ^factor encoded within the T3SS-encoding region of *P. syringae *[59]. We therefore used a Hidden Markov Model (HMM) trained using 38 known HrpL-regulated genes of *P. syringae *pv *tomato *DC3000 to mine the genome of WH6 for *hrp*-boxes [56,60].

We found 115 *hrp*-boxes in the genome of WH6 (bit score ≥ 3.0) but only 24 were within 500 bp of a CDS. Two were located upstream of *rspF *and *rscR *in the T3SS-encoding region, with bit scores of 7.9 and 3.2, respectively. We also identified a *hrp*-box upstream of *rspJ *but it had a lower bit score of 1.2. Fifteen of the CDSs downstream of candidate *hrp*-boxes had annotated functions not typically associated with type III effectors and we did not list them as possible candidates (data not shown). The remaining eight CDSs downstream of *hrp*-boxes were annotated as hypothetical proteins and the five with the highest bit scores for their corresponding *hrp*-boxes were not present in the genomes of Pf-5 and Pf0-1; all but PFWH6_1942 were unique to WH6 (Table 4). Further investigation of their first amino-terminal residues indicated that three have characteristics suggestive of T3SS-dependent secretion [56,61,62].

**Table 4:** Putative *hrp*-boxes and candidate type III effector genes in WH6

Bit Score	*hrp*-box sequence	CDS*	Distance from *hrp*-box	Translocation Signal^
7.9	TGGAACTGAATAGCCAGTACTGACCAC	*rspF*	26	-

6.7	AGGAACCGATTCCGACAGATGGGCCAC	1942	77	A, B

5.3	CGGAACCTTTACCGGCACCTGAACACT	2917	248	A, B, C

5.1	TGGAACGAAATCGTCGATCAAACCACT	3173	58	A, C

5	TGGAACCGTATTGCGTAAGACGTCACT	1252	82	-

4.3	GGGAACCGCATCGGTTGCCTTCCAAC	3940	56	-

3.6	TGAAACCGGCACGGCGTGCCTGACCCT	*rscR*	324	A

1.2	TGGAACCAGGTGGGCGGGGCTTGCCAC	*rspJ*	33	A, B

Only coding sequences with no predicted homology or identifiable orthologs in Pf-5 or Pf0-1 are shown.

*PFWH6_# unless otherwise noted; ^N-terminal translocation signal scores were assigned based on "A"; >10% serine in first 50 amino acids, "B"; absence of aliphatic amino acids in position 3 or 4, and/or "C"; absence of negatively-charged amino acids in the first 12 amino acids [62].

Our two computational approaches yielded very few candidate type III effectors. One possible explanation is that because RspL and HrpL have only 50% identity (70% similarity), they recognize slightly different cis-regulatory sequences and our HMM was not adequately trained for the cis-regulatory sequence recognized by RspL. This is an unlikely scenario. Three sequences with strong similarity to the *hrp*-box of *P. syringae *were found in the T3SS-encoding regions for WH6 and SBW25 [15]. Additionally, it has been observed that all HrpL-dependent phytopathogenic bacteria share an identical motif in the *hrp*-box despite having as little as 52% similarity [63]. Furthermore, in σ^70 ^factors, DNA binding specificity is conferred by the helix-turn-helix domain 4.2 [64,65]. Domain 4.2 of the WH6 RspL is highly similar (82.5%) to the corresponding domain of HrpL. An alternative explanation is that WH6 encodes very few type III effectors with little homology to those that have been identified. This is not unheard of. *P. aeruginosa *for example, has only three type III effectors [66,67].

### Type VI secretion systems

The type VI secretion system (T6SS) is another secretion apparatus that is common to host-associated bacteria. Computational approaches suggest the T6SS may also be in *P. fluorescens *[68,69]. We found evidence for two complete and functional T6SSs in WH6. We have named these two systems T6SS-1 (Figure 6b; PFWH6_5796-5812) and T6SS-2 (Figure 6c; PFWH6_3251-3270). It is not uncommon for organisms to possess multiple T6SSs that are of different lineages and acquired independently [68]. Additionally, in other strains that have been characterized, different T6SSs appear to be independently regulated, suggesting each T6SS may have functions specific to different aspects of the lifestyle of the bacterium [70]. Whether this is also the case with WH6 awaits further characterization.

T6SS-1 belongs to the group A lineage and shares homology and synteny to HSI-I of *P. aeruginosa *PAO1 [68]. We therefore named the corresponding genes in WH6 according to the nomenclature established for HSI-I (Figure 6b). Synteny extended beyond the T6SS-encoding region and included the *tagQRST *genes bordering *ppkA*. We did not, however, find evidence for *tagJ1 *in WH6 [68,71]. T6SS-2 is a group B secretion system [68]. Less is understood about the group B secretion systems but T6SS-2 showed strong homology and synteny to a corresponding T6SS encoded in the genome of the phytopathogen *P. syringae *pv *tomato *DC3000 (Figure 6c; [28]).

There are few proteins that are demonstrable type VI effectors. Three homologs of VgrG and Hcp have been shown to be secreted by the T6SS but both likely have functions for the T6SS itself [72-74]. We found four *vgrG *genes, of which only one was associated with T6SS-1. The other three genes were found elsewhere in the genome. Whether products from these latter three are secreted proteins of the T6SS or merely homologous in sequence is unknown. Both T6SSs of WH6 had a homolog of *hcp*. Recently, three additional proteins from *P. aeruginosa *PAO1 were shown to be secreted by the T6SS, but their orthologs were not found in WH6 [75].

## Conclusions

*P. fluorescens *is a genetically and physiologically diverse species found in many habitats. We sequenced the genome of the isolate WH6 because it produces Germination-Arrest Factor (GAF), an herbicide that specifically arrests seed germination of graminaceous species. Comparisons of the WH6 genome to genomes of SBW25, Pf-5, and Pf0-1 helped better define this species, with WH6 and SBW25 forming one lineage. Comparative studies revealing substantial similarity in gene inventory and synteny supported its placement and the argument of at least two major lineages of *P. fluorescens *[5].

With the genome sequence, we were able to deduce potential functions for two genes necessary for GAF activity. One encoded a candidate anti-sigma factor. Our previous results suggest that PrtR is an activator and suggests it has a role in regulating expression of genes necessary for GAF. The second gene encoded a candidate aminotransferase, which tentatively supports our previous speculation that GAF is a small peptide or amino acid analog. Further studies are required to confirm their functions. A less labor-intensive and saturating screen will also be necessary for a fuller understanding of the pathway controlling GAF expression and biosynthesis. The genome sequence will certainly facilitate such future endeavors.

We also identified a number of mechanisms that potentially affect plant health and some typically associated with host-associated bacteria. One of the more extensively characterized mechanisms is the type III secretion system. WH6 appears to encode the necessary repertoire of genes for a complete and functional T3SS. We also identified two T6SSs in WH6. Further studies are necessary to identify the role these secretion systems and their effectors play in the lifestyle of WH6.

## Methods

### Sequencing DNA flanking Tn5-insertions

To determine the sites of Tn*5 *insertion, genomic DNA from the two GAF mutants, WH6-2::Tn*5 *and WH6-3::Tn*5 *was digested with *Bam*HI or *Pst*I, respectively. We used Southern blotting with a biotinylated probe of the Tet^R ^gene from pUTmini-Tn*5*gfp to identify the fusion fragments between the Tet^R ^gene and flanking WH6 DNA [76]. DNA fragments of corresponding size were cloned into pBluescript SK+ (Stratagene, La Jolla, CA), transformed into *E. coli *DH5∝, selected based on tetracycline resistance, isolated, and sequenced outwards using primers to the Tet^R ^gene.

### *P. fluorescens *WH6 Genome Sequencing

We used the ZR Fungal/Bacterial DNA Kit to isolate genomic DNA from *P. fluorescens *WH6 grown overnight in LB at 28°C (Zymo Research, Orange, CA). Purity and concentration were determined using a Nanodrop ND1000 (Thermo Scientific, Waltham, MA). For Illumina-based sequencing, we prepared the DNA according to the instructions of the manufacturer and sequenced the DNA fragments on the Illumina GA I and II using 36-cycle (4 channels) and paired-end 76-cycle (1 channel) sequencing, respectively (Illumina, San Diego, CA). The Sanger and Illumina sequencing was done at the Center for Genome Research and Biocomputing Core Labs (CGRB; Oregon State University, Corvallis, OR). We also sequenced genomic DNA using the 454 FLX GS LR70 (454, Branford, CT). Preparation and sequencing by 454 was done at the Consortium for Comparative Genomics (University of Colorado Health Sciences Center, Denver, CO).

### Short-read assembly

For Illumina-derived reads, the last four and six bases were trimmed from the 36 mer and 76 mer reads, respectively. We filtered out all Illumina-derived short reads that had ambiguous bases. For the paired-end reads, both reads were filtered out if one read of a pair had ambiguous bases. We used Velvet 0.7.55 to *de novo *assemble the reads [22]. We assembled short reads from the different sequencing platforms independently, as well as in combination. We wrote *ad hoc *shell scripts to test different Velvet parameters of hash length, coverage cutoff, and expected coverage. In total, we generated approximately 75 different genome assemblies of WH6. Shell scripts are available for download (http://changlab.cgrb.oregonstate.edu).

### Improvements to the high-quality draft assembly

We developed *ad hoc *Perl scripts to use BLASTN to compare between each of the WH6 assemblies and used congruency in contigs from the various assemblies to cull those with potential misassemblies (see next section for description of scripts; data were visualized using blast_draw.pl). We used Tablet 1.10.01.28 to inspect the remaining genome assemblies for depth of coverage and potential misassemblies [77]. Finally, we used Mauve Aligner 2.3 and the genome sequence of *P. fluorescens *SBW25 as a reference to reorder WH6 contigs greater than 100 bp from our assembly with highest confidence [9,23]. Default settings were used for Mauve Aligner 2.3.

### Physical and sequence gap closure

To identify contigs that potentially flanked a physical gap, we wrote and used Contig_end_blast_A.pl, to extract 300 bp of sequence from the ends of each contig greater than one kb in size and use the contig ends as queries in a BLASTN search against the NCBI nt database. We also wrote and used Contig_end_blast_B.pl to find contig ends that shared significant homology (e-value ≤ 0.02) to the same reference sequence but aligned to different regions no more than one kb apart. The contigs corresponding to these ends were thus predicted to be physically linked in the genome of WH6. PCR using contig-specific primers and subsequent Sanger sequencing were used to close the physical gaps (See Additional file 2: Table S2). To correct the sizes for sequence gaps larger than 300 bp, we used a similar approach. PCR was used to validate our corrections for sequence gaps.

Contig_end_blast_A.pl, Contig_end_blast_B.pl, and blast_draw.pl are available for download from our website at: http://changlab.cgrb.oregonstate.edu.

### Genome Annotation

We used a custom pipeline to annotate the improved high-quality draft assembly of WH6 as previously described [78]. The only exceptions were that we used Glimmer 3.02 rather than Glimmer 2 to predict coding regions and gene models were trained using the "long-orfs" option ([79]; http://www.cbcb.umd.edu/software/glimmer/).

### Bioinformatic analyses

For analysis of synteny, we first parsed the genomes of SBW25, Pf-5 and Pf0-1 into all possible 25 mers and identified their unique 25 mer sequences. Next, we used CASHX to align all unique 25 mers from each of three genomes to both strands of a formatted database from the WH6 genome sequence [80]. Only perfect matches were allowed. We identified the corresponding genome coordinates for each 25 mer and the matching 25 mer in the WH6 genome and used R to plot the start coordinates of each matching pair in an XY graph [81].

Phylogenomic relationships were determined using HAL ([27]; http://aftol.org/pages/Halweb3.htm). HAL uses an all-by-all reciprocal BLASTP to create a similarity matrix from e-values. These are then used to group proteins into related clusters using a Markov Clustering algorithm. Clusters containing one protein sequence from each genome that identified each other as best hits were extracted, concatenated within each proteome, and used to infer phylogenetic relationships. Phylogenetic trees were visualized using the Archaeopteryx & Forester Java application ([82]; http://www.phylosoft.org/archaeopteryx/).

Hidden Markov Models (HMMs) for *hrp*-boxes were trained from a set of 38 confirmed *hrp*-boxes in the *P. syringae *pv *tomato *DC3000 genome [28,56,57,60]. The HMM for the extracytoplasmic function σ^70 ^factors was downloaded from http://www.g2l.bio.uni-goettingen.de/software/f_software.html. Searches were done using HMMER 2.3.2 (http://hmmer.janelia.org/).

Circular diagrams were plotted using DNAplotter ([83]; http://www.sanger.ac.uk/Software/ Artemis/circular/).

## Authors' contributions

JAK prepared the DNA for sequencing, assembled and analyzed the genome sequence of WH6, as well as drafted the manuscript. SAG annotated the genome. ABH sequenced the DNA flanking the Tn*5 *insertions, did the preliminary analyses of gene functions, and helped draft the manuscript. ALC assisted with analyzing the different assemblies. DIM, GMB, DJA, and JHC conceived of the study and drafted the manuscript. All authors read and approved the final manuscript.

## Supplementary Material

Additional file 1 **Table S1: *De novo *assembled contigs of WH6 that are candidates for physical gap closure**. Click here for file

Additional file 2 **Table S2: Sequences of oligonucleotides used to close physical gaps**. Click here for file

## References

[B1] Haas D Defago G Biological control of soil-borne pathogens by fluorescent pseudomonads Nat Rev Microbiol 2005 3 4 307 319 10.1038/nrmicro1129 15759041

[B2] Flores-Vargas RD O'Hara GW Isolation and characterization of rhizosphere bacteria with potential for biological control of weeds in vineyards J Appl Microbiol 2006 100 5 946 954 10.1111/j.1365-2672.2006.02851.x 16629995

[B3] Li Y Sun A Zhuang X Xu L Chen S Li M Research progress on microbial herbicides Crop Protection 2003 22 247 252 10.1016/S0261-2194(02)00189-8

[B4] Armstrong D Azevedo M Mills D Bailey B Russell B Groenig A Halgren A Banowetz G McPhail K Germination-Arrest Factor (GAF): 3. Determination that the herbicidal activity of GAF is associated with a ninhydrin-reactive compound and counteracted by selected amino acids Biological Control 2009 51 1 181 190 10.1016/j.biocontrol.2009.06.004

[B5] Yamamoto S Kasai H Arnold DL Jackson RW Vivian A Harayama S Phylogeny of the genus *Pseudomonas*: intrageneric structure reconstructed from the nucleotide sequences of *gyrB *and *rpoD *genes Microbiology 2000 146 Pt 10 2385 2394 11021915 10.1099/00221287-146-10-2385

[B6] Medini D Donati C Tettelin H Masignani V Rappuoli R The microbial pan-genome Curr Opin Genet Dev 2005 15 6 589 594 10.1016/j.gde.2005.09.006 16185861

[B7] Tettelin H Riley D Cattuto C Medini D Comparative genomics: the bacterial pan-genome Curr Opin Microbiol 2008 11 5 472 477 10.1016/j.mib.2008.09.006 19086349

[B8] Paulsen IT Press CM Ravel J Kobayashi DY Myers GS Mavrodi DV DeBoy RT Seshadri R Ren Q Madupu R Dodson RJ Durkin AS Brinkac LM Daugherty SC Sullivan SA Rosovitz MJ Gwinn ML Zhou L Schneider DJ Cartinhour SW Nelson WC Weidman J Watkins K Tran K Khouri H Pierson EA Pierson LS Thomashow LS Loper JE Complete genome sequence of the plant commensal *Pseudomonas fluorescens *Pf-5 Nat Biotechnol 2005 23 7 873 878 10.1038/nbt1110 15980861 PMC7416659

[B9] Silby MW Cerdeno-Tarraga AM Vernikos GS Giddens SR Jackson RW Preston GM Zhang XX Moon CD Gehrig SM Godfrey SA Knight CG Malone JG Robinson Z Spiers AJ Harris S Challis GL Yaxley AM Harris D Seeger K Murphy L Rutter S Squares R Quail MA Saunders E Mavromatis K Brettin TS Bentley SD Hothersall J Stephens E Thomas CM Parkhill J Levy SB Rainey PB Thomson NR Genomic and genetic analyses of diversity and plant interactions of *Pseudomonas fluorescens* Genome Biol 2009 10 5 R51 10.1186/gb-2009-10-5-r51 19432983 PMC2718517

[B10] Merhej V Royer-Carenzi M Pontarotti P Raoult D Massive comparative genomic analysis reveals convergent evolution of specialized bacteria Biol Direct 2009 4 13 10.1186/1745-6150-4-13 19361336 PMC2688493

[B11] Galan JE Wolf-Watz H Protein delivery into eukaryotic cells by type III secretion machines Nature 2006 444 7119 567 573 10.1038/nature05272 17136086

[B12] Grant SR Fisher EJ Chang JH Mole BM Dangl JL Subterfuge and manipulation: type III effector proteins of phytopathogenic bacteria Annu Rev Microbiol 2006 60 425 449 10.1146/annurev.micro.60.080805.142251 16753033

[B13] Jones JD Dangl JL The plant immune system Nature 2006 444 7117 323 329 10.1038/nature05286 17108957

[B14] Rezzonico F Defago G Moenne-Loccoz Y Comparison of ATPase-encoding type III secretion system *hrcN *genes in biocontrol fluorescent Pseudomonads and in phytopathogenic proteobacteria Appl Environ Microbiol 2004 70 9 5119 5131 10.1128/AEM.70.9.5119-5131.2004 15345390 PMC520869

[B15] Preston GM Bertrand N Rainey PB Type III secretion in plant growth-promoting *Pseudomonas fluorescens *SBW25 Mol Microbiol 2001 41 5 999 1014 10.1046/j.1365-2958.2001.02560.x 11555282

[B16] Jackson RW Preston GM Rainey PB Genetic characterization of *Pseudomonas fluorescens *SBW25 *rsp *gene expression in the phytosphere and *in vitro* J Bacteriol 2005 187 24 8477 8488 10.1128/JB.187.24.8477-8488.2005 16321952 PMC1317024

[B17] Ideses D Gophna U Paitan Y Chaudhuri RR Pallen MJ Ron EZ A degenerate type III secretion system from septicemic *Escherichia coli *contributes to pathogenesis J Bacteriol 2005 187 23 8164 8171 10.1128/JB.187.23.8164-8171.2005 16291689 PMC1291271

[B18] Rezzonico F Binder C Defago G Moenne-Loccoz Y The type III secretion system of biocontrol *Pseudomonas fluorescens *KD targets the phytopathogenic Chromista *Pythium ultimum *and promotes cucumber protection Mol Plant Microbe Interact 2005 18 9 991 1001 10.1094/MPMI-18-0991 16167769

[B19] Banowetz GM Azevedo MD Armstrong DJ Halgren AB Mills DI Germination-Arrest Factor (GAF): Biological properties of a novel, naturally-occurring herbicide produced by selected isolates of rhizosphere bacteria Biological Control 2008 46 3 380 390 10.1016/j.biocontrol.2008.04.016

[B20] Banowetz GM Azevedo MD Armstrong DJ Mills DI Germination arrest factor (GAF): Part 2. Physical and chemical properties of a novel, naturally occurring herbicide produced by *Pseudomonas fluorescens *WH6 Biological Control 2009 50 103 110 10.1016/j.biocontrol.2009.03.011

[B21] Chain PS Grafham DV Fulton RS Fitzgerald MG Hostetler J Muzny D Ali J Birren B Bruce DC Buhay C Cole JR Ding Y Dugan S Field D Garrity GM Gibbs R Graves T Han CS Harrison SH Highlander S Hugenholtz P Khouri HM Kodira CD Kolker E Kyrpides NC Lang D Lapidus A Malfatti SA Markowitz V Metha T Nelson KE Parkhill J Pitluck S Qin X Read TD Schmutz J Sozhamannan S Sterk P Strausberg RL Sutton G Thomson NR Tiedje JM Weinstock G Wollam A Detter JC Genomics. Genome project standards in a new era of sequencing Science 2009 326 5950 236 237 10.1126/science.1180614 19815760 PMC3854948

[B22] Zerbino DR Birney E Velvet: algorithms for *de novo *short read assembly using de Bruijn graphs Genome Res 2008 18 5 821 829 10.1101/gr.074492.107 18349386 PMC2336801

[B23] Rissman AI Mau B Biehl BS Darling AE Glasner JD Perna NT Reordering contigs of draft genomes using the Mauve aligner Bioinformatics 2009 25 16 2071 2073 10.1093/bioinformatics/btp356 19515959 PMC2723005

[B24] Pop M Salzberg SL Bioinformatics challenges of new sequencing technology Trends Genet 2008 24 3 142 149 18262676 10.1016/j.tig.2007.12.007 PMC2680276

[B25] Gross H Loper JE Genomics of secondary metabolite production by *Pseudomonas *spp Nat Prod Rep 2009 26 11 1408 1446 10.1039/b817075b 19844639

[B26] Wilkinson B Micklefield J Chapter 14. Biosynthesis of nonribosomal peptide precursors Methods Enzymol 2009 458 353 378 full_text 19374990 10.1016/S0076-6879(09)04814-9

[B27] Robbertse B Reeves JB Schoch CL Spatafora JW A phylogenomic analysis of the Ascomycota Fungal Genet Biol 2006 43 10 715 725 10.1016/j.fgb.2006.05.001 16781175

[B28] Buell CR Joardar V Lindeberg M Selengut J Paulsen IT Gwinn ML Dodson RJ Deboy RT Durkin AS Kolonay JF Madupu R Daugherty S Brinkac L Beanan MJ Haft DH Nelson WC Davidsen T Zafar N Zhou L Liu J Yuan Q Khouri H Fedorova N Tran B Russell D Berry K Utterback T Van Aken SE Feldblyum TV D'Ascenzo M Deng WL Ramos AR Alfano JR Cartinhour S Chatterjee AK Delaney TP Lazarowitz SG Martin GB Schneider DJ Tang X Bender CL White O Fraser CM Collmer A The complete genome sequence of the Arabidopsis and tomato pathogen *Pseudomonas syringae *pv. *tomato *DC3000 Proc Natl Acad Sci USA 2003 100 18 10181 10186 10.1073/pnas.1731982100 12928499 PMC193536

[B29] Feil H Feil WS Chain P Larimer F DiBartolo G Copeland A Lykidis A Trong S Nolan M Goltsman E Thiel J Malfatti S Loper JE Lapidus A Detter JC Land M Richardson PM Kyrpides NC Ivanova N Lindow SE Comparison of the complete genome sequences of *Pseudomonas syringae *pv. *syringae *B728a and pv. *tomato *DC3000 Proc Natl Acad Sci USA 2005 102 31 11064 11069 10.1073/pnas.0504930102 16043691 PMC1182459

[B30] Stover CK Pham XQ Erwin AL Mizoguchi SD Warrener P Hickey MJ Brinkman FS Hufnagle WO Kowalik DJ Lagrou M Garber RL Goltry L Tolentino E Westbrock-Wadman S Yuan Y Brody LL Coulter SN Folger KR Kas A Larbig K Lim R Smith K Spencer D Wong GK Wu Z Paulsen IT Reizer J Saier MH Hancock RE Lory S Olson MV Complete genome sequence of *Pseudomonas aeruginosa *PAO1, an opportunistic pathogen Nature 2000 406 6799 959 964 10.1038/35023079 10984043

[B31] Joardar V Lindeberg M Jackson RW Selengut J Dodson R Brinkac LM Daugherty SC Deboy R Durkin AS Giglio MG Madupu R Nelson WC Rosovitz MJ Sullivan S Crabtree J Creasy T Davidsen T Haft DH Zafar N Zhou L Halpin R Holley T Khouri H Feldblyum T White O Fraser CM Chatterjee AK Cartinhour S Schneider DJ Mansfield J Collmer A Buell CR Whole-genome sequence analysis of *Pseudomonas syringae *pv. *phaseolicola *1448A reveals divergence among pathovars in genes involved in virulence and transposition J Bacteriol 2005 187 18 6488 6498 10.1128/JB.187.18.6488-6498.2005 16159782 PMC1236638

[B32] Studholme DJ Ibanez SG MacLean D Dangl JL Chang JH Rathjen JP A draft genome sequence and functional screen reveals the repertoire of type III secreted proteins of *Pseudomonas syringae *pathovar *tabaci *11528 BMC Genomics 2009 10 395 10.1186/1471-2164-10-395 19703286 PMC2745422

[B33] Arakawa K Tomita M The GC Skew Index: A Measure of Genomic Compositional Asymmetry and the Degree of Replicational Selection Evol Bioinform Online 2007 3 159 168 19461976 PMC2684130

[B34] Rocha EP The replication-related organization of bacterial genomes Microbiology 2004 150 Pt 6 1609 1627 10.1099/mic.0.26974-0 15184548

[B35] Parkhill J Sebaihia M Preston A Murphy LD Thomson N Harris DE Holden MT Churcher CM Bentley SD Mungall KL Cerdeno-Tarraga AM Temple L James K Harris B Quail MA Achtman M Atkin R Baker S Basham D Bason N Cherevach I Chillingworth T Collins M Cronin A Davis P Doggett J Feltwell T Goble A Hamlin N Hauser H Holroyd S Jagels K Leather S Moule S Norberczak H O'Neil S Ormond D Price C Rabbinowitsch E Rutter S Sanders M Saunders D Seeger K Sharp S Simmonds M Skelton J Squares R Squares S Stevens K Unwin L Whitehead S Barrell BG Maskell DJ Comparative analysis of the genome sequences of *Bordetella pertussis*, *Bordetella parapertussis *and *Bordetella bronchiseptica* Nat Genet 2003 35 1 32 40 10.1038/ng1227 12910271

[B36] Law CJ Maloney PC Wang DN Ins and outs of major facilitator superfamily antiporters Annu Rev Microbiol 2008 62 289 305 10.1146/annurev.micro.61.080706.093329 18537473 PMC2612782

[B37] Blanvillain S Meyer D Boulanger A Lautier M Guynet C Denance N Vasse J Lauber E Arlat M Plant carbohydrate scavenging through tonB-dependent receptors: a feature shared by phytopathogenic and aquatic bacteria PLoS One 2007 2 2 e224 10.1371/journal.pone.0000224 17311090 PMC1790865

[B38] Tock MR Dryden DT The biology of restriction and anti-restriction Curr Opin Microbiol 2005 8 4 466 472 10.1016/j.mib.2005.06.003 15979932

[B39] Burger M Woods RG McCarthy C Beacham IR Temperature regulation of protease in *Pseudomonas fluorescens *LS107d2 by an ECF sigma factor and a transmembrane activator Microbiology 2000 146 Pt 12 3149 3155 11101673 10.1099/00221287-146-12-3149

[B40] Hughes KT Mathee K The anti-sigma factors Annu Rev Microbiol 1998 52 231 286 10.1146/annurev.micro.52.1.231 9891799

[B41] Yoshimura T Jhee KH Soda K Stereospecificity for the hydrogen transfer and molecular evolution of pyridoxal enzymes Biosci Biotechnol Biochem 1996 60 2 181 187 10.1271/bbb.60.181 9063963

[B42] Marchler-Bauer A Anderson JB Chitsaz F Derbyshire MK DeWeese-Scott C Fong JH Geer LY Geer RC Gonzales NR Gwadz M He S Hurwitz DI Jackson JD Ke Z Lanczycki CJ Liebert CA Liu C Lu F Lu S Marchler GH Mullokandov M Song JS Tasneem A Thanki N Yamashita RA Zhang D Zhang N Bryant SH CDD: specific functional annotation with the Conserved Domain Database Nucleic Acids Res 2009 37 Database D205 210 10.1093/nar/gkn845 18984618 PMC2686570

[B43] Staron A Sofia HJ Dietrich S Ulrich LE Liesegang H Mascher T The third pillar of bacterial signal transduction: classification of the extracytoplasmic function (ECF) sigma factor protein family Mol Microbiol 2009 74 3 557 581 10.1111/j.1365-2958.2009.06870.x 19737356

[B44] Lindeberg M Myers CR Collmer A Schneider DJ Roadmap to new virulence determinants in *Pseudomonas syringae*: insights from comparative genomics and genome organization Mol Plant Microbe Interact 2008 21 6 685 700 10.1094/MPMI-21-6-0685 18624633

[B45] Bogdanove AJ Beer SV Bonas U Boucher CA Collmer A Coplin DL Cornelis GR Huang HC Hutcheson SW Panopoulos NJ Van Gijsegem F Unified nomenclature for broadly conserved *hrp *genes of phytopathogenic bacteria Mol Microbiol 1996 20 3 681 683 10.1046/j.1365-2958.1996.5731077.x 8736546

[B46] Hutcheson SW Bretz J Sussan T Jin S Pak K Enhancer-binding proteins HrpR and HrpS interact to regulate *hrp*-encoded type III protein secretion in *Pseudomonas syringae *strains J Bacteriol 2001 183 19 5589 5598 10.1128/JB.183.19.5589-5598.2001 11544221 PMC95450

[B47] Wei Z Kim JF Beer SV Regulation of *hrp *genes and type III protein secretion in *Erwinia amylovora *by HrpX/HrpY, a novel two-component system, and HrpS Mol Plant Microbe Interact 2000 13 11 1251 1262 10.1094/MPMI.2000.13.11.1251 11059492

[B48] Alfano JR Bauer DW Milos TM Collmer A Analysis of the role of the *Pseudomonas syringae *pv. *syringae *HrpZ harpin in elicitation of the hypersensitive response in tobacco using functionally non-polar *hrpZ *deletion mutations, truncated HrpZ fragments, and *hrmA *mutations Mol Microbiol 1996 19 4 715 728 10.1046/j.1365-2958.1996.415946.x 8820642

[B49] Ortiz-Martin I Thwaites R Mansfield JW Beuzon CR Negative regulation of the Hrp type III secretion system in *Pseudomonas syringae *pv. *phaseolicola* Mol Plant Microbe Interact 2010 23 5 682 701 10.1094/MPMI-23-5-0682 20367475

[B50] Preston G Deng WL Huang HC Collmer A Negative regulation of *hrp *genes in *Pseudomonas syringae *by HrpV J Bacteriol 1998 180 17 4532 4537 9721292 10.1128/jb.180.17.4532-4537.1998 PMC107464

[B51] Deng WL Preston G Collmer A Chang CJ Huang HC Characterization of the *hrpC *and *hrpRS *operons of *Pseudomonas syringae *pathovars *syringae*, *tomato*, and *glycinea *and analysis of the ability of *hrpF*, *hrpG*, *hrcC*, *hrpT*, and *hrpV *mutants to elicit the hypersensitive response and disease in plants J Bacteriol 1998 180 17 4523 4531 9721291 10.1128/jb.180.17.4523-4531.1998 PMC107463

[B52] Ramos AR Morello JE Ravindran S Deng WL Huang HC Collmer A Identification of *Pseudomonas syringae *pv. *syringae *61 type III secretion system Hrp proteins that can travel the type III pathway and contribute to the translocation of effector proteins into plant cells J Bacteriol 2007 189 15 5773 5778 10.1128/JB.00435-07 17526708 PMC1951817

[B53] Knodler LA Vallance BA Hensel M Jackel D Finlay BB Steele-Mortimer O *Salmonella *type III effectors PipB and PipB2 are targeted to detergent-resistant microdomains on internal host cell membranes Mol Microbiol 2003 49 3 685 704 10.1046/j.1365-2958.2003.03598.x 12864852

[B54] Jelenska J Yao N Vinatzer BA Wright CM Brodsky JL Greenberg JT A J domain virulence effector of *Pseudomonas syringae *remodels host chloroplasts and suppresses defenses Curr Biol 2007 17 6 499 508 10.1016/j.cub.2007.02.028 17350264 PMC1857343

[B55] Vinatzer BA Jelenska J Greenberg JT Bioinformatics correctly identifies many type III secretion substrates in the plant pathogen *Pseudomonas syringae *and the biocontrol isolate *P. fluorescens *SBW25 Mol Plant Microbe Interact 2005 18 8 877 888 10.1094/MPMI-18-0877 16134900

[B56] Chang JH Urbach JM Law TF Arnold LW Hu A Gombar S Grant SR Ausubel FM Dangl JL A high-throughput, near-saturating screen for type III effector genes from *Pseudomonas syringae* Proc Natl Acad Sci USA 2005 102 7 2549 2554 10.1073/pnas.0409660102 15701698 PMC549004

[B57] Ferreira AO Myers CR Gordon JS Martin GB Vencato M Collmer A Wehling MD Alfano JR Moreno-Hagelsieb G Lamboy WF DeClerck G Schneider DJ Cartinhour SW Whole-genome expression profiling defines the HrpL regulon of *Pseudomonas syringae *pv. *tomato *DC3000, allows *de novo *reconstruction of the *Hrp *cis clement, and identifies novel coregulated genes Mol Plant Microbe Interact 2006 19 11 1167 1179 10.1094/MPMI-19-1167 17073300

[B58] Fouts DE Abramovitch RB Alfano JR Baldo AM Buell CR Cartinhour S Chatterjee AK D'Ascenzo M Gwinn ML Lazarowitz SG Lin NC Martin GB Rehm AH Schneider DJ van Dijk K Tang X Collmer A Genomewide identification of *Pseudomonas syringae *pv. *tomato *DC3000 promoters controlled by the HrpL alternative sigma factor Proc Natl Acad Sci USA 2002 99 4 2275 2280 10.1073/pnas.032514099 11854524 PMC122355

[B59] Innes RW Bent AF Kunkel BN Bisgrove SR Staskawicz BJ Molecular analysis of avirulence gene *avrRpt2 *and identification of a putative regulatory sequence common to all known *Pseudomonas syringae *avirulence genes J Bacteriol 1993 175 15 4859 4869 8335641 10.1128/jb.175.15.4859-4869.1993 PMC204939

[B60] Schechter LM Vencato M Jordan KL Schneider SE Schneider DJ Collmer A Multiple approaches to a complete inventory of *Pseudomonas syringae *pv. *tomato *DC3000 type III secretion system effector proteins Mol Plant Microbe Interact 2006 19 11 1180 1192 10.1094/MPMI-19-1180 17073301

[B61] Guttman DS Vinatzer BA Sarkar SF Ranall MV Kettler G Greenberg JT A functional screen for the type III (Hrp) secretome of the plant pathogen *Pseudomonas syringae* Science 2002 295 5560 1722 1726 10.1126/science.295.5560.1722 11872842

[B62] Petnicki-Ocwieja T Schneider DJ Tam VC Chancey ST Shan L Jamir Y Schechter LM Janes MD Buell CR Tang X Collmer A Alfano JR Genomewide identification of proteins secreted by the Hrp type III protein secretion system of *Pseudomonas syringae *pv. *tomato *DC3000 Proc Natl Acad Sci USA 2002 99 11 7652 7657 10.1073/pnas.112183899 12032338 PMC124312

[B63] Nissan G Manulis S Weinthal DM Sessa G Barash I Analysis of promoters recognized by HrpL, an alternative sigma-factor protein from *Pantoea agglomerans *pv. *gypsophilae* Mol Plant Microbe Interact 2005 18 7 634 643 10.1094/MPMI-18-0634 16042009

[B64] Harley CB Reynolds RP Analysis of *E. coli *promoter sequences Nucleic Acids Res 1987 15 5 2343 2361 10.1093/nar/15.5.2343 3550697 PMC340638

[B65] Potvin E Sanschagrin F Levesque RC Sigma factors in *Pseudomonas aeruginosa* FEMS Microbiol Rev 2008 32 1 38 55 10.1111/j.1574-6976.2007.00092.x 18070067

[B66] Feltman H Schulert G Khan S Jain M Peterson L Hauser AR Prevalence of type III secretion genes in clinical and environmental isolates of *Pseudomonas aeruginosa* Microbiology 2001 147 Pt 10 2659 2669 11577145 10.1099/00221287-147-10-2659

[B67] Wolfgang MC Kulasekara BR Liang X Boyd D Wu K Yang Q Miyada CG Lory S Conservation of genome content and virulence determinants among clinical and environmental isolates of *Pseudomonas aeruginosa* Proc Natl Acad Sci USA 2003 100 14 8484 8489 10.1073/pnas.0832438100 12815109 PMC166255

[B68] Bingle LE Bailey CM Pallen MJ Type VI secretion: a beginner's guide Curr Opin Microbiol 2008 11 1 3 8 10.1016/j.mib.2008.01.006 18289922

[B69] Shrivastava S Mande SS Identification and functional characterization of gene components of Type VI Secretion system in bacterial genomes PLoS One 2008 3 8 e2955 10.1371/journal.pone.0002955 18698408 PMC2492809

[B70] Mougous JD Cuff ME Raunser S Shen A Zhou M Gifford CA Goodman AL Joachimiak G Ordonez CL Lory S Walz T Joachimiak A Mekalanos JJ A virulence locus of *Pseudomonas aeruginosa *encodes a protein secretion apparatus Science 2006 312 5779 1526 1530 10.1126/science.1128393 16763151 PMC2800167

[B71] Hsu F Schwarz S Mougous JD TagR promotes PpkA-catalysed type VI secretion activation in *Pseudomonas aeruginosa* Mol Microbiol 2009 72 5 1111 1125 10.1111/j.1365-2958.2009.06701.x 19400797 PMC3402362

[B72] Pukatzki S Ma AT Revel AT Sturtevant D Mekalanos JJ Type VI secretion system translocates a phage tail spike-like protein into target cells where it cross-links actin Proc Natl Acad Sci USA 2007 104 39 15508 15513 10.1073/pnas.0706532104 17873062 PMC2000545

[B73] Pukatzki S Ma AT Sturtevant D Krastins B Sarracino D Nelson WC Heidelberg JF Mekalanos JJ Identification of a conserved bacterial protein secretion system in *Vibrio cholerae *using the *Dictyostelium *host model system Proc Natl Acad Sci USA 2006 103 5 1528 1533 10.1073/pnas.0510322103 16432199 PMC1345711

[B74] Wu HY Chung PC Shih HW Wen SR Lai EM Secretome analysis uncovers an Hcp-family protein secreted via a type VI secretion system in *Agrobacterium tumefaciens* J Bacteriol 2008 190 8 2841 2850 10.1128/JB.01775-07 18263727 PMC2293243

[B75] Hood RD Singh P Hsu F Guvener T Carl MA Trinidad RR Silverman JM Ohlson BB Hicks KG Plemel RL Li M Schwarz S Wang WY Merz AJ Goodlett DR Mougous JD A type VI secretion system of *Pseudomonas aeruginosa *targets a toxin to bacteria Cell Host Microbe 2010 7 1 25 37 10.1016/j.chom.2009.12.007 20114026 PMC2831478

[B76] Matthysse AG Stretton S Dandie C McClure NC Goodman AE Construction of GFP vectors for use in Gram-negative bacteria other than *Escherichia coli* FEMS Microbiol Lett 1996 145 1 87 94 10.1111/j.1574-6968.1996.tb08561.x 8931331

[B77] Milne I Bayer M Cardle L Shaw P Stephen G Wright F Marshall D Tablet--next generation sequence assembly visualization Bioinformatics 2010 26 3 401 402 10.1093/bioinformatics/btp666 19965881 PMC2815658

[B78] Giovannoni SJ Hayakawa DH Tripp HJ Stingl U Givan SA Cho JC Oh HM Kitner JB Vergin KL Rappe MS The small genome of an abundant coastal ocean methylotroph Environ Microbiol 2008 10 7 1771 1782 10.1111/j.1462-2920.2008.01598.x 18393994

[B79] Delcher AL Harmon D Kasif S White O Salzberg SL Improved microbial gene identification with GLIMMER Nucleic Acids Res 1999 27 23 4636 4641 10.1093/nar/27.23.4636 10556321 PMC148753

[B80] Fahlgren N Sullivan CM Kasschau KD Chapman EJ Cumbie JS Montgomery TA Gilbert SD Dasenko M Backman TW Givan SA Carrington JC Computational and analytical framework for small RNA profiling by high-throughput sequencing Rna 2009 15 5 992 1002 10.1261/rna.1473809 19307293 PMC2673065

[B81] R Development Core Team R: A language and environment for statistical computing 2007 R Foundation for Statistical Computing. Vienna, Austria http://www.R-project.org ISBN 3-900051-07-0

[B82] Zmasek CM Eddy SR ATV: display and manipulation of annotated phylogenetic trees Bioinformatics 2001 17 4 383 384 10.1093/bioinformatics/17.4.383 11301314

[B83] Carver T Thomson N Bleasby A Berriman M Parkhill J DNAPlotter: circular and linear interactive genome visualization Bioinformatics 2009 25 1 119 120 10.1093/bioinformatics/btn578 18990721 PMC2612626

[B84] Alfano JR Charkowski AO Deng WL Badel JL Petnicki-Ocwieja T van Dijk K Collmer A The *Pseudomonas syringae Hrp *pathogenicity island has a tripartite mosaic structure composed of a cluster of type III secretion genes bounded by exchangeable effector and conserved effector loci that contribute to parasitic fitness and pathogenicity in plants Proc Natl Acad Sci USA 2000 97 9 4856 4861 10.1073/pnas.97.9.4856 10781092 PMC18322

[B85] Chen C Beattie GA Characterization of the osmoprotectant transporter OpuC from *Pseudomonas syringae *and demonstration that cystathionine-beta-synthase domains are required for its osmoregulatory function J Bacteriol 2007 189 19 6901 6912 10.1128/JB.00763-07 17660277 PMC2045199

[B86] Chen C Beattie GA *Pseudomonas syringae *BetT is a low-affinity choline transporter that is responsible for superior osmoprotection by choline over glycine betaine J Bacteriol 2008 190 8 2717 2725 10.1128/JB.01585-07 18156257 PMC2293270

[B87] Tan Y Donovan WP Deletion of *aprA *and *nprA *genes for alkaline protease A and neutral protease A from *Bacillus thuringiensis*: effect on insecticidal crystal proteins J Biotechnol 2001 84 1 67 72 10.1016/S0168-1656(00)00328-X 11035189

[B88] Bowen D Rocheleau TA Blackburn M Andreev O Golubeva E Bhartia R ffrench-Constant RH Insecticidal toxins from the bacterium *Photorhabdus luminescens* Science 1998 280 5372 2129 2132 10.1126/science.280.5372.2129 9641921

[B89] Lee JS Heo YJ Lee JK Cho YH KatA, the major catalase, is critical for osmoprotection and virulence in *Pseudomonas aeruginosa *PA14 Infect Immun 2005 73 7 4399 4403 10.1128/IAI.73.7.4399-4403.2005 15972537 PMC1168586

[B90] Yu J Penaloza-Vazquez A Chakrabarty AM Bender CL Involvement of the exopolysaccharide alginate in the virulence and epiphytic fitness of *Pseudomonas syringae *pv. *syringae* Mol Microbiol 1999 33 4 712 720 10.1046/j.1365-2958.1999.01516.x 10447881

[B91] Hettwer U Jaeckel FR Boch J Meyer M Rudolph K Ullrich MS Cloning, nucleotide sequence, and expression in *Escherichia coli *of levansucrase genes from the plant pathogens *Pseudomonas syringae *pv. *glycinea *and *P. syringae *pv. *phaseolicola* Appl Environ Microbiol 1998 64 9 3180 3187 9726857 10.1128/aem.64.9.3180-3187.1998 PMC106707

[B92] Koczan JM McGrath MJ Zhao Y Sundin GW Contribution of *Erwinia amylovora *exopolysaccharides amylovoran and levan to biofilm formation: implications in pathogenicity Phytopathology 2009 99 11 1237 1244 10.1094/PHYTO-99-11-1237 19821727

[B93] Ellison DW Miller VL Regulation of virulence by members of the MarR/SlyA family Curr Opin Microbiol 2006 9 2 153 159 10.1016/j.mib.2006.02.003 16529980

[B94] Johnson TL Abendroth J Hol WG Sandkvist M Type II secretion: from structure to function FEMS Microbiol Lett 2006 255 2 175 186 10.1111/j.1574-6968.2006.00102.x 16448494

[B95] Meyers DJ Berk RS Characterization of phospholipase C from *Pseudomonas aeruginosa *as a potent inflammatory agent Infect Immun 1990 58 3 659 666 2106492 10.1128/iai.58.3.659-666.1990 PMC258516

[B96] Bronstein PA Marrichi M Cartinhour S Schneider DJ DeLisa MP Identification of a twin-arginine translocation system in *Pseudomonas syringae *pv. *tomato *DC3000 and its contribution to pathogenicity and fitness J Bacteriol 2005 187 24 8450 8461 10.1128/JB.187.24.8450-8461.2005 16321949 PMC1317023

[B97] Caldelari I Mann S Crooks C Palmer T The Tat pathway of the plant pathogen *Pseudomonas syringae *is required for optimal virulence Mol Plant Microbe Interact 2006 19 2 200 212 10.1094/MPMI-19-0200 16529382

[B98] Arrebola E Cazorla FM Codina JC Gutierrez-Barranquero JA Perez-Garcia A de Vicente A Contribution of mangotoxin to the virulence and epiphytic fitness of *Pseudomonas syringae *pv. *syringae* Int Microbiol 2009 12 2 87 95 19784928

